# A DIVERSE LIFE IS A BETTER LIFE? THE ASSOCIATION BETWEEN ACTIVITY DIVERSITY AND PSYCHOLOGICAL WELL-BEING IN HONG KONG

**DOI:** 10.1093/geroni/igad104.1184

**Published:** 2023-12-21

**Authors:** Zhixuan Lin, Helene Fung

**Affiliations:** The Chinese University of Hong Kong, Hong Kong, Hong Kong; The Chinese University of Hong Kong, Hong Kong, Hong Kong

## Abstract

The debate between the devotedness and evenness of activity participation has long existed. On the one hand, the theory of selective optimization with compensation (Baltes & Baltes, 1990) argues that successful aging depends on concentrating one’s participation on fewer domains of activities. On the other hand, the social integration perspective (Rosow, 1967) posits that diversity indicates extensive knowledge and a sense of purpose, which contributes to well-being. A previous study found a positive relationship between activity diversity and older adults’ psychological well-being in a U.S. sample (Lee et al., 2018). However, it might not be the case for people living in extremely hot weather as diverse activities might increase the risk of exposure to the heat. The current study examines their relationship in the hot summer months in Hong Kong, a tropical Asian city. Community-dwelling participants aged 60 years or above (N = 344, Mage = 67.15, SDage = 5.26) completed surveys 3 times each day over 10 days, reporting their activities, positive and negative affect, meaning and engagement of each activity, and loneliness. The regional heat-risk index was acquired from Hua et al. (2021). Results showed that activity diversity negatively correlated with positive affect, meaningfulness and engagement of daily activities, and positively correlated with loneliness. Heat risk did not moderate the relationship, but it correlated with less positive and more negative affect. Our findings suggest that Hong Kong older adults benefit from focused rather than broad participation in activities.

